# Transformation of Oil Palm Waste-Derived Cellulose into Solid Polymer Electrolytes: Investigating the Crucial Role of Plasticizers

**DOI:** 10.3390/polym13213685

**Published:** 2021-10-26

**Authors:** Cheyma Naceur Abouloula, Muhammad. Rizwan, Vidhya Selvanathan, Rosiyah Yahya, Khaled Althubeiti, Hend I. Alkhammash, Md. Akhtaruzzaman, A. Oueriagli

**Affiliations:** 1 MEE Lab, Faculty of Science Semlalia, University of Cadi Ayyad, Marrakesh 40090, Morocco; oueriagli@uca.ac.ma; 2 Department of Chemistry, The University of Lahore, Lahore 54000, Pakistan; rizi_chem1981@hotmail.com; 3 Solar Energy Research Institute (SERI), Universiti Kebangsaan Malaysia (UKM), Bangi 43600, Malaysia; vidhya@ukm.edu.my; 4 Department of Chemistry, Faculty of Science, University of Malaya, Kuala Lumpur 50603, Malaysia; rosiyah@um.edu.my; 5 Department of Chemistry, College of Science, Taif University, Taif 21944, Saudi Arabia; k.althubeiti@tu.edu.sa; 6 Department of Electrical Engineering, College of Engineering, Taif University, Taif 21944, Saudi Arabia; Khamash.h@tu.edu.sa

**Keywords:** carboxymethyl cellulose, glycerol, polymer electrolyte, ionic conductivity

## Abstract

This study explores the possibility of transforming lignocellulose-rich agricultural waste materials into value-added products. Cellulose was extracted from an empty fruit bunch of oil palm and further modified into carboxymethyl cellulose (CMC), a water-soluble cellulose derivative. The CMC was then employed as the polymeric content in fabrication of solid polymer electrolyte (SPE) films incorporated with lithium iodide. To enhance the ionic conductivity of the solid polymer electrolytes, the compositions were optimized with different amounts of glycerol as a plasticizing agent. The chemical and physical effects of plasticizer content on the film composition were studied by Fourier transform infrared (FTIR) and X-ray diffraction (XRD) analysis. FTIR and XRD analysis confirmed the interaction plasticizer with the polymer matrix and the amorphous nature of fabricated SPEs. The highest ionic conductivity of 6.26 × 10^−2^ S/cm was obtained with the addition of 25 wt % of glycerol. By fabricating solid polymer electrolytes from oil palm waste-derived cellulose, the sustainability of the materials can be retained while reducing the dependence on fossil fuel-derived materials in electrochemical devices.

## 1. Introduction

Malaysia is one of the world’s leading producers of palm oil, giving rise to the production of enormous amounts of agricultural waste from the oil palm industry. For every 100 tonnes of fresh oil palm fruit bunches processed, about 22 tonnes of crude palm oil and 26 tonnes of empty fruit bunches are produced. These oil palm wastes are rich in cellulose, a natural polymer with excellent mechanical properties which can be exploited for various material developments. One of such novel applications is the employment of cellulose as the polymeric material for fabrication of solid polymer electrolyte (SPE) films. SPEs are commonly used in various electrochemical devices such as in batteries [1], electrochromic devices [2,3], supercapacitors [4,5] and solar cells [6]. By far, most commercially available SPE are made up of petroleum-derived synthetic polymers. By substituting the non-renewable material with oil palm-derived cellulose, the ecological footprint of the product can be reduced while simultaneously converting the biomass into a useful component.

Since cellulose in its native state is practically insoluble in most solvents, the polymer is often chemically modified to soluble derivatives such as carboxymethyl cellulose (CMC). Upon functionalization of the cellulose, it can be easily converted into conductive films by adding inorganic salts. Previously, cellulose derivatives-based SPEs have been exploited for fabrication of SPEs. For instance, Selvakumar and co-workers have used cellulose acetate (CA) as a biodegradable polymer electrolyte for supercapacitors. The electrochemical properties and degradation tests of CA-based SPEs were investigated, then used for the fabrication of supercapacitors that showed good capacitive nature and stability during cycling [7].

The application of biopolymerbased SPE is restricted by its low ionic conductivity at ambient temperature. Hence, most of the studies are dedicated to enhancing the ionic conductivity of the films by various strategies including blending with other polymers, insertion of fillers, and plasticizers. Amongst these methods, the use of plasticizer to produce higher ionic conductivity has shown appreciable results in recent research. To enhance the ionic conductivity of SPEs, it was found that the choice of a good plasticizer with a low molecular weight and high dielectric constant or polar solvent, such as propylene carbonate, ethylene carbonate, is highly effective [8,9]. The addition of a plasticizer leads to an increase in the amorphousness of polymer electrolyte and dissociate ion pairs into free cations and anions, thereby leading to an overall enhancement in conductivity. A high plasticizer/salt ratio modifies the morphological, thermal, and electrochemical performance of the matrix, which also leads to the enhancement of conductivity. Chinnam et al. have prepared SPEs by mixing methyl cellulose, LiClO_4_, and polyethylene glycol (PEG), to be used as a binder for Li-ion batteries and replace the commonly used materials, i.e., polyvinylidene fluoride–hexafluoropropylene (PVDF–HFP) or polyvinylidene fluoride (PVDF). The fabricated SPE showed good electrochemical stability and interfacial resistance with an optimum ambient conductivity of 1.6 × 10^−5^ S/cm [10].

Although previous literature has established the prospects of cellulose as polymer host in fabrication of SPE films, most of these studies have employed commercially available cellulose powder. Like any other natural polymer, the physical and chemical properties of cellulose films depend on the source from which they are derived. Thus, to fully exploit agrowaste-derived cellulose as a precursor material for SPE application, it is essential to ensure that the films are fabricated from the waste material itself. There are numerous studies on fabrication of cellulose films from different agricultural by-products including sugarcane bagasse [11], pineapple peel [12], *Ficus natalensis* bark cloth [13], rice husks [14] and corn husks [15]. However, research focussing on agrowaste-derived cellulose for SPE fabrication have been scarce. More importantly, the electrical performance of such waste-based SPE are poor, disabling their application into electrochemical devices. For instance, carboxymethyl cellulose film fabricated from coconut husk recorded ionic conductivity of 4.82 × 10^−4^ S/cm [16]. Similarly, biopolymer electrolytes comprised of kenaf fibre-derived CMC and ammonium acetate achieved the highest conductivity at ambient temperature of 5.77 × 10^−4^ S/cm [17].

Therefore, this study is dedicated to explore the potential of elevating the electrical conductivity of oil palm waste fibre-derived cellulose films. To achieve this, two main strategies were adopted. Firstly, the extracted cellulose chemically transformed into carboxymethyl cellulose. Our previous study has verified that the CMC derivative is essential to impart solubility and diminish high crystallinity of the native oil palm-based cellulose. However, the ionic conductivity of lithium iodide-incorporated CMC films can only achieve 5.58 × 10^−3^ S/cm. In this work, glycerol was infused as a plasticizer to improve the film’s conductivity. Different amounts of glycerol were added to the polymer electrolyte matrix to identify the best composition ratio with good physico-chemical properties and high ionic conductivity for potential use in electrochemical devices.

## 2. Materials and Methods 

### 2.1. Materials

Raw fibres of EFB oil palm were collected from Malaysian Agricultural Research and Development Institute (MARDI) (Selangor, Malaysia). NaOH, glacial acetic acid, monochloroacetic acid, and isopropanol were supplied by Merck (Darmstadt, Germany). Sodium chlorite, ethanol, methanol, glycerol, and lithium iodide were purchased from Sigma-Aldrich Co. (Saint Louis, MO, USA). The used chemicals were of analytical grade and were used without further purification.

### 2.2. Extraction Cellulose from Oil Palm Waste

The extraction of cellulose from raw EFB oil palm fibres is comprised of two steps, as reported in our previous work [18]. Firstly, the fibres were treated in an aqueous solution of 4 wt % of NaOH at 80 °C for 3 h. Then, the solid was filtered and washed several times with distilled water. The process was repeated thrice. This was followed by bleaching in equal parts of acetate buffer, aqueous chlorite (1.7% *w*/*v*), and distilled water, at 120 °C for 4 h. The bleaching treatment was repeated twice. 

### 2.3. Carboxymethylation of Cellulose

The extracted cellulose powder was dispersed in isopropanol followed by the addition of 30% *w*/*v* solution of NaOH and kept under stirring for 30 min at room temperature. Monochloroacetic acid was added to the suspension and stirred for 90 min, then was allowed to stand for 1.5 h at 55 °C. The mixture was carefully separated, suspended in methanol and neutralized using glacial acetic acid before being washed several times with ethanol, followed by absolute methanol. The solid was dried overnight at 50 °C. 

### 2.4. Fabrication of CMC-Based Solid Polymer Electrolyte

**Figure 1:**
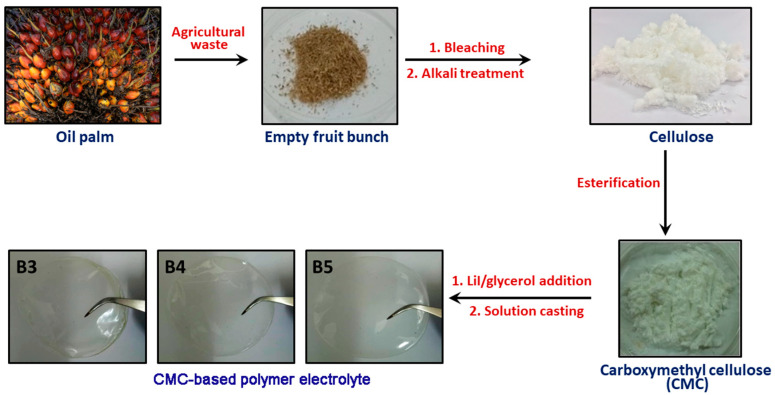
Transformation of oil palm waste into solid polymer electrolyte films.

The plasticized films were fabricated by preparing an aqueous solution containing dissolved CMC, LiI and glycerol. For every film, 0.2 g of CMC was mixed with 0.13 g of LiI, as per the optimized composition of LiI from our previously reported work [11]. Different amounts of glycerol, ranging from 5 to 30% (as outlined in Table 1) were added into the aqueous CMC-LiI solution while stirring, until a homogenous solution was formed. The solutions were cast on petri dishes separately, dried at room temperature, and kept in a desiccator. Figure 1 depicts the formation of plasticized CMC-based SPEs from oil palm-derived cellulose. 

### 2.5. Characterization

A PerkinElmer Spotlight 400 FTIR spectrometer (UK) via the ATR technique was used to examine the structural and chemical changes induced during the extraction and modification of the cellulose. The samples were analysed at a resolution of 2 cm^−1^ and 32 scans within a spectral range of 650 to 4000 cm^−1^. X-ray diffraction measurements were conducted using an Empyrean diffractometer (PANalytical, Almelo, Netherlands). The diffracted intensity of the Cu Kα radiation was measured at 2*θ* angle between 10° and 60° with a step size of 0.026°. The crystallinity index (*CrI*) was determined by an empirical method using the following equation:

(1) $$CrI\;\left(\%\right)=\frac{I_{002}-I_{am}}{I_{002}}\times 100$$

where *I*_002_ is the maximum intensity of the (002) lattice diffraction peak and *I_am_* is the intensity scattered by the amorphous part of the sample. The diffraction peak for plane (002) is located at a diffraction angle of around 2*θ* = 22.5°, and the intensity scattered by the amorphous part was measured as the low intensity at a diffraction angle of around 2*θ* = 18.0°. Electrochemical impedance spectroscopy (EIS) was used to measure the ionic conductivity of CMC-LiI-based film using a 3532-50 LCR Hi-Tester, Hioki (Nagano, Japan), with a frequency range from 50 Hz to 5 MHz and a temperature varying from 303 to 353 K. The ionic conductivity (*σ*) was calculated using the following equation:

(2) $$\sigma =t/\left(R_{b}\times A\right)$$

where *t* is the thickness of the sample measured, *R_b_* is the bulk resistance of the sample, and *A* is the contact surface area. All the EIS measurements were repeated three times for each sample, and the average value was calculated.

## 3. Results and Discussion

### 3.1. Synthesis of Carboxymethyl Cellulose from Oil Palm Waste

CMC was synthesized by reacting cellulose and monochloroacetic acid in an alkali environment. The yield % of the obtained CMC was calculated based on the theoretical amount of reacted cellulose with the carboxymethyl group following the equation:

(3) $$Yield\;\%\;of\;CMC=\frac{Weight\;of\;CMC\;obtained\;\left(g\right)}{Theoretical\;weight\;of\;CMC\;\left(g\right)}\times 100$$

The yield % of CMC calculated following the above equation was found to be 84.84%. The chemical identity of the cellulose extracted from oil palm waste and the CMC derivative was confirmed via NMR, XRD, and DSC analysis in our previous publication [18].

**Figure 2:**
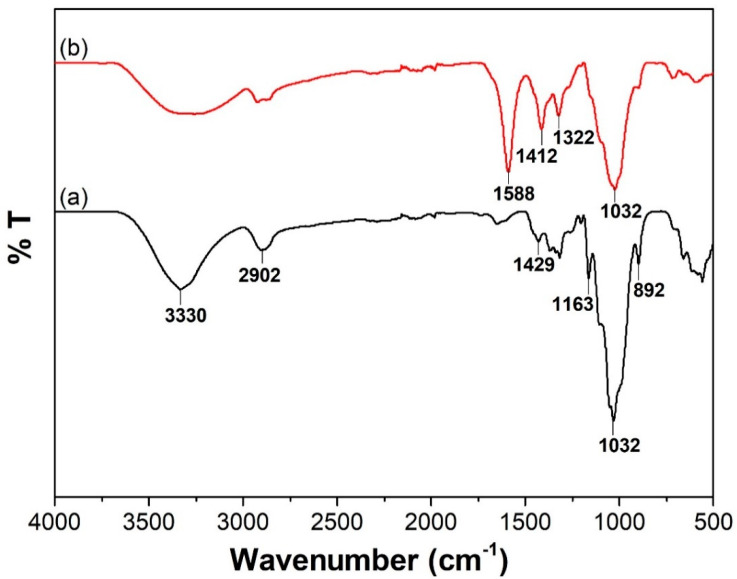
FTIR spectra of (**a**) cellulose extracted from oil palm waste and (**b**) carboxymethyl cellulose.

The purity of cellulose extracted from oil palm fibres was verified by FTIR analysis of the sample, as shown in Figure 2a. The bands between 1000 and 1500 cm^−1^ correspond to characteristic absorption of cellulose. The bands at 1429 cm^−1^, 1163 cm^−1^, 1032 cm^−1^, and 892 cm^−1^ are attributed to C-H bending and C-O-C stretching of β-linked glucose subunits. Upon carboxymethylation, a significant absorption band appeared at 1588 cm^−1^, which is assigned to the carboxyl group, thus verifying the transformation of cellulose into CMC (Figure 2b).

### 3.2. Chemical Interactions within the Polymer Film

**Figure 3:**
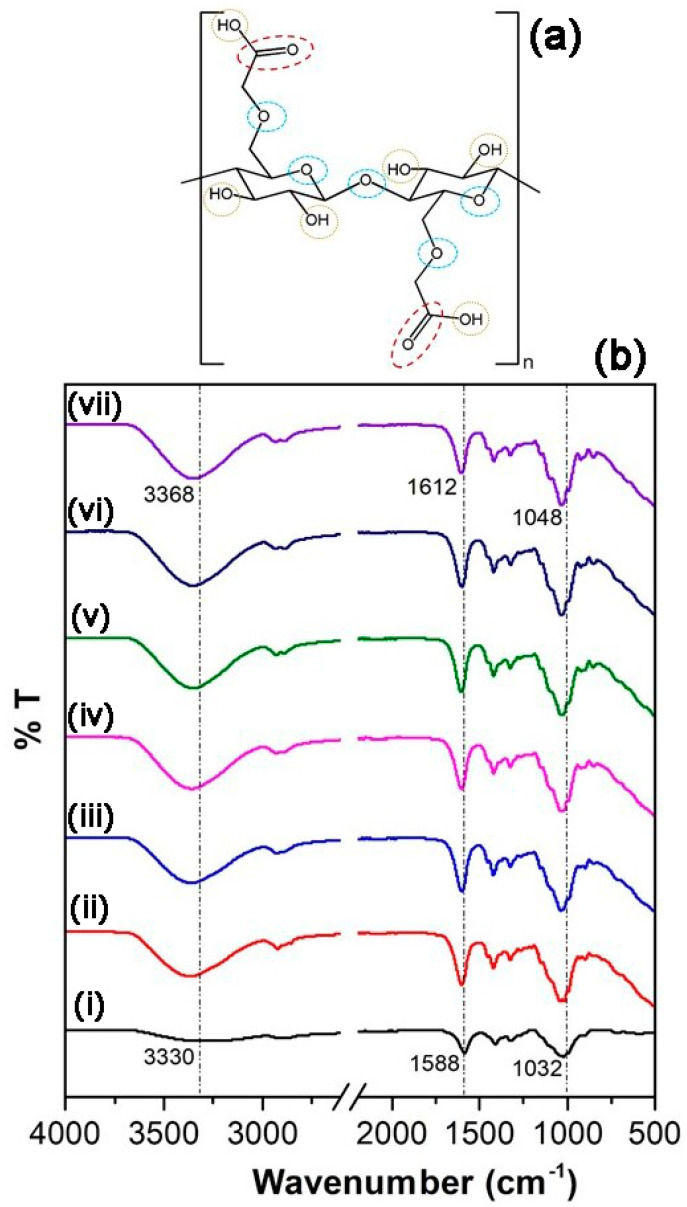
(**a**) Chemical scheme of possible complexation site in the polymer chain and (**b**) FTIR spectra of (i) CMC powder and CMC-LiI films with (ii) 5%, (iii) 10%, (iv) 15%, (v) 20%, (vi) 25%, and (vii) 30% glycerol content.

The FTIR spectra of plasticized SPEs in Figure 3 show appreciable changes in absorbance with changing proportion of glycerol in the composition of SPEs. The scheme in Figure 3 outlines the possible site of interactions between the polymer chains and the plasticizer molecules and the ionic species present with the electrolyte matrix. Evidently, significant changes in the FTIR spectra of the polymer films were observed for absorption bands corresponding to hydroxyl, carboxyl, and ether groups of the CMC backbone, indicating possible complexation at these sites.

The addition of glycerol affected the O-H stretching vibration of SPEs, which shifted from 3330 cm^−1^ in CMC to 3368 cm^−1^ in the films. This is expected primarily due to the hydrogen bonding interaction between the hydroxyls in glycerol and in the polysaccharide chains. The presence of glycerol also affected the carboxyl group absorption at 1588 cm^−1^, causing an upshift to 1612 cm^−1^. The stretching vibrations of C-O in the glucose subunits experienced an upshift from 1032 cm^−1^ to 1048 cm^−1^. It is evident that the main chemical interactions between the polymer and the plasticizer occur within an oxygen-associated functional group such as (-COO, -OH,-C-O), which serve as chemically active sites, as explained by Liu et al. [19]. The trend in the FTIR spectra upon plasticization tallies with previously reported cellulose-based films [20,21].

### 3.3. Crystallinity Analysis

**Figure 4:**
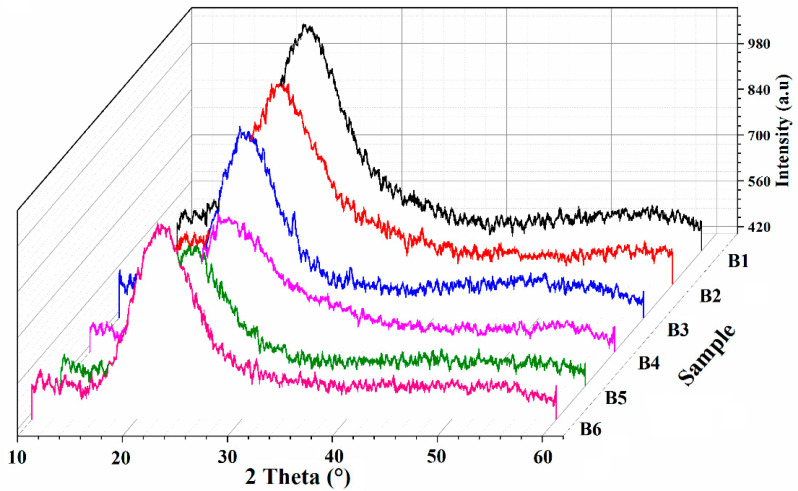
X–ray diffractograms of CMC–LiI–Gly-based SPEs.

The addition of glycerol is complemented with modification in the crystallinity of the polymer chains within the SPEs, as shown in Figure 4. The increase of glycerol in fabricated SPEs leads to a wider peak and a decrease of its intensity to reach a maximum with the addition of 25% of glycerol content. These changes indicate that the addition of glycerol helps to increase the amorphousness of the plasticized SPEs membrane [22]. The decline in crystallinity is further confirmed by degree of crystallinity calculation, as tabulated in Table 2, which shows a downward trend up to 25% glycerol. Beyond 25% of glycerol, the crystallinity of CMC-LiI-Gly SPEs increases, which can be explained as the impact of plasticizer overloading, leading to phase separation between the different components.

### 3.4. Conductivity Analysis

To obtain better results and enhance ionic conductivity of CMC-LiI SPEs, glycerol was added as a plasticizer owing to its high dielectric constant ε_r_ = 41.14 [23]. The addition of plasticizer to the SPEs does not supply more ions, instead, it facilitates more dissociation of salt and generates separate conducting pathways for the migration of free ions. 

**Figure 5:**
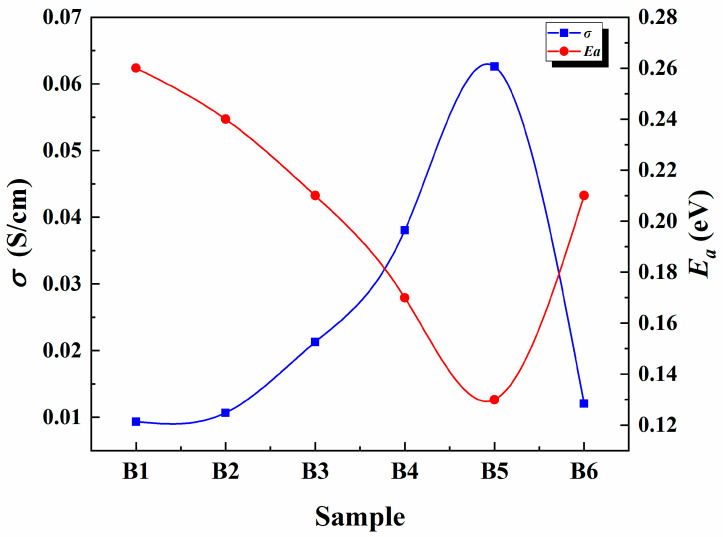
The trend in the ionic conductivity with different amounts of glycerol.

The ionic conductivity of CMC-LiI SPEs was improved by adding glycerol as a liquid plasticizer, and the highest value (6.26 × 10^−2^ S/cm) was achieved with the addition of 25% glycerol (Figure 5). This increase is due to the high dielectric constant of glycerol and its low molecular weight, which modify the physical properties of SPEs [24]. Beyond 25% of glycerol content, the ionic conductivity dramatically decreased. This decrease may be due to the bulk quantity of the plasticizer, which promotes the polymer/plasticizer phase separation, an observation that is in agreement with the increased crystallinity of the film [25].

Interestingly, the ionic conductivity values recorded in this work are better than previously reported commercial cellulose-based films and are on par with synthetic polymer-based SPEs, as shown in Table 3. This, in fact, consolidates that, with appropriate chemical modifications, cellulose derivatives of agricultural waste origin can be tailored to form functional materials with desirable physico-chemical properties.

The SPE film fabricated in this work attains an excellent ionic conductivity value, thus enabling the application of the films in various electrochemical devices. In recent times, biobased solid polymer electrolytes with the conductivity of magnitude within 10^−3^ and 10^−2^ S/cm have been proven to show good performance upon incorporation into full devices. For instance, incorporation of cellulose acetate and ammonium nitrate in different ratios were designed to prepare eco-friendly biopolymer electrolytes and the highest ionic conductivity of 1.02 × 10^−3^ S/cm was obtained [30]. The film with the highest ionic conductivity was used in the development of a primary proton battery and proton exchange membrane fuel cell. Monisha and co-workers found that the performances of the fabricated biopolymer electrolyte were optimum and could replace the standard Nafion 117 membrane. In another work, cellulose triacetate, poly(polyethylene glycol methacrylate) and ionic liquid were combined to make a solid polymer electrolyte and showed a high ionic conductivity of 5.24 × 10^−3^ S/cm [31]. The fabricated solid polymer electrolytes were tested on a Li cell and were found to be highly stable up to 5 V and safer with high performance for lithium batteries. Promising solid polymer electrolytes have been also prepared by blending methylcellulose and chitosan doped with ammonium thiocyanate for electrochemical double-layer capacitor (EDLC) application [32]. The electrolyte with the highest DC conductivity of 2.81 × 10^−3^ S/cm was used as an electrode separator in EDLC and exhibited good performance. Hence, the oil palm-derived CMC films incorporated with glycerol and lithium iodide have prospects to be used as a biodegradable SPE in place of conventional synthetic polymer-based films.

However, to fully realize the potential of the films, it is vital to thoroughly investigate the thermal and chemical stability of the films in the future. In addition to that, examining physical parameters such as tensile strength, flexibility, adhesiveness, and hydrophilicity will be useful to improve the integration of the films as SPE in electrochemical devices. 

### 3.5. Temperature-Dependent Conductivity Analysis

**Figure 6:**
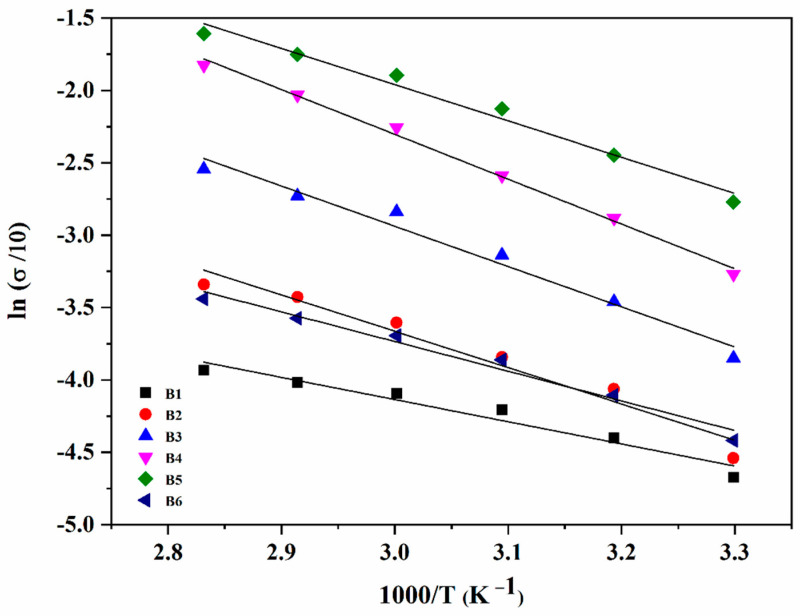
The trend in the ionic conductivity with different amounts of glycerol.

Practically, the fabricated SPEs are expected to be exposed to high temperature. Thus, it is vital to evaluate the temperature-dependent ionic conductivity values of plasticized SPEs. Figure 6 shows the ionic conductivity of the SPEs with the increase of temperature from 30 °C to 80 °C. The ionic conductivity increases linearly with temperature. This linear behaviour shows that there is no phase transition in the polymer matrix within that temperature range [24]. Since the conductivity behaviour at elevated temperature obeys the Arrhenius equation, the activation energy, *E_a_*, was calculated using the following equation:

(4) $$\sigma =\sigma_{0}\exp\left[-\frac{E_{a}}{kT}\right]$$

where *σ*_0_ is the pre-exponential factor, *E_a_* is the activation energy of ionic conduction, *k* is the Boltzmann constant and *T* is the temperature in Kelvin. 

Based on the calculation, the *E_a_* was found to be in the range of 0.18–0.22 eV, which reflects the amorphous nature of the SPEs. The increase in ionic conductivity behaviour of SPEs with the rise of temperature is due to the dislocation and migration of ions across the SPEs. Furthermore, the heat provides more energy for the segmental motion of polymer chains, and thus facilitates ions transportation. The increase in conductivity can be translated by the increase of ions motion and chain flexibility of polymers due to the rise of temperature.

The Anderson–Stuart model defined that an ion can move freely in the matrix if it has energy to free itself from its site and enough energy for its migration [33,34]. Thus, the activation energy is the main factor in deciding the ionic conductivity at a particular temperature for a specific system. With the increase of glycerol, *E_a_* decreases up to a minimum with the addition of 25 wt %, followed by an increase of its energy. Figure 5 clearly shows that the ionic conductivity and *E_a_* have an inverse relationship, where the conductivity is maximum with minimum value of *E_a_* at 65% salt and 25 wt % plasticizer. This result can be attributed to the significant amorphous nature of the film with the given salt and plasticizer contents, as evident from XRD, facilitating the ion migration through the movement of chains in the amorphous domain [35]. Selvasekarapandian and co-workers reported that the density of ions in the electrolyte increases with the increase in salt or glycerol content. Thus, the energy barrier for the proton transport decreases, leading to a decrease in the *E_a_* value. These results are in agreement with the values obtained in this work.

## 4. Conclusions

The CMC-LiI-based SPEs plasticized with glycerol were successfully fabricated using a solution-cast method. The interactions between polymer, salt, and plasticizer were investigated by FTIR spectroscopy. The XRD showed that the amorphousness of the SPEs facilitates the ions movement, which reflected the increase of ionic conductivity. The optimum ionic conductivity was found to be 6.26 × 10^−2^ S/cm with a composition of 25 wt % of glycerol. This translates the appreciable effect of the addition of glycerol as a plasticizer. Thus, the plasticized SPEs can be used in electrochemical devices.

## Figures and Tables

**Table 1:** Composition of plasticized SPEs with various masses of glycerol.

Designations	CMC (g)	LiI (g)	Glycerol	
			(g)	*w*/*w* (%)
B1	0.2	0.13	0.02	5
B2			0.03	10
B3			0.05	15
B4			0.07	20
B5			0.08	25
B6			0.10	30

**Table 2:** Degree of crystallinity of plasticized SPEs with various mass of glycerol.

Sample	% Glycerol (*w*/*w*)	Degree of Crystallinity (%)
CMC powder	-	36.9
B1	5	25.0
B2	10	22.9
B3	15	20.1
B4	20	18.3
B5	25	17.6
B6	30	23.8

**Table 3:** Recent literature on solid polymer electrolytes.

Polymeric Content	Salt	Additives	Ionic Conductivity (S cm^−1^)	Reference
Carboxymethyl cellulose	Lithium tetrafluoroborate	Glycerol	0.0037	[24]
Carboxylated cellulose	Lithium hexafluorophosphate	Ethylene carbonate + dimethyl carbonate + ethyl methyl carbonate	0.0018	[26]
Carboxymethyl cellulose	-	Polyaniline	0.018	[27]
Poly(ethylene oxide)	Sodium iodide	1-methyl-3-propylimidazolium iodide	0.0094	[28]
Poly(vinylidene fluoride-hexafluoro propylene	-	1-ethyl-3-methylimidazolium tetrafluoroborate + graphene oxide	0.025	[29]
Carboxymethyl cellulose	Lithium iodide	Glycerol	0.063	This study

## Data Availability

Not applicable.
